# A Preliminary Report on Brain-Derived Extracellular Vesicle as Novel Blood Biomarkers for Sport-Related Concussions

**DOI:** 10.3389/fneur.2018.00239

**Published:** 2018-04-12

**Authors:** Keisuke Kawata, Masato Mitsuhashi, Randy Aldret

**Affiliations:** ^1^Department of Kinesiology, School of Public Health-Bloomington, Indiana University, Bloomington, IN, United States; ^2^NanoSomiX Inc., Aliso Viejo, CA, United States; ^3^School of Kinesiology, University of Louisiana at Lafayette, Lafayette, LA, United States

**Keywords:** mild traumatic brain injury, concussion, neurotrauma, ice hockey, subconcussion, microvesicle

## Abstract

The purpose of the study was to test the utility of unique panel of blood biomarkers as a means to reflect one’s recovery process after sport-related neurotrauma. We established a panel of biomarkers that reacted positive with CD81 (extracellular vesicle marker) and various neuron- and glia-specific antigens [e.g., neurofilament light polypeptide (NF-L), tau, synaptosome-associated protein 25 (SNAP25), glial fibrillary acidic protein, and myelin basic protein]. We first evaluated test–retest reliabilities of brain-derived exosome markers, followed by an application of these markers in eight professional ice hockey players to detect cumulative neuronal burden from a single ice hockey season. During the season, two players were diagnosed with concussions by team physician based on an exhibition of symptoms as well as abnormality in balance and ocular motor testing. One player reached symptom-free status 7 days after the concussion, while the other player required 36 days for symptoms to completely resolve. Blood samples and clinical assessments including balance error scoring system and near point of convergence throughout recovery process were obtained. Biomarkers indicative of axonal damage, neuronal inflammation, and glial activation showed excellent test–retest reliabilities (intraclass correlation coefficient: 0.713–0.998, *p*’s < 0.01). There was a statistically significant increase in the NF-L marker at post-season follow-up compared to pre-season baseline (*Z* = −2.100, *P* = 0.036); however the statistical significance did not withstand Bonferroni correction for multiple comparisons. In concussion cases, neuronal and microglia markers notably increased after concussions, with the unique expression patterns being similar to that of concussion recovery process. These longitudinal data coupled with excellent test–retest reliabilities of novel array of blood biomarkers potentially reflect the damage in neural cell structures and metabolic crisis due to concussion. However, future studies with larger sample size and appropriate control groups to evaluate sensitivity and specificity of these markers are needed. This preliminary case report suggests the potential utility of multimodal blood biomarkers for concussion prognosis and recovery assessment.

## Introduction

Mild traumatic brain injury (mTBI) presenting with negative intracranial bleeding, often referred to as concussion, is the most common neurological injury. The majority of patients with concussion eliciting symptoms (e.g., headache, dizziness, and light sensitivity) and behavioral abnormality (e.g., balance and eye tracking) recover within a 7- to 14-day period, while approximately 15% of patients exhibit lingering signs and symptoms beyond 2 weeks (1, 2). Outward symptoms are thought to be the most direct method to evaluate individual’s well-being. However, emerging evidence indicates that neural cell damage and healing process do not correlate with symptom scores, such that, microstructural damage and functional impairment persist even after complete resolution of concussion-related symptoms (3–5). Therefore, an objective panel of assessments that are highly sensitive and specific to the extent of cellular damage is needed for “at risk” individuals such as athletes and military service members.

The use of blood biomarkers is known to be a promising avenue as they enable the detection of subtle cellular, structural, and metabolic changes after neural damage (6, 7). Elevations in protein-based biomarkers including tau protein, neurofilament light polypeptide (NF-L), glial fibrillary acidic protein (GFAP), S100-beta, *a*II-Spectrin N-terminal fragment, and ubiquitin C-terminal hydrolase L1 have been observed in concussion patients across ages, injury mechanisms, and occupations, indicating their immense utility in concussion diagnosis (8–15). These markers are predominantly expressed by brain parenchymal cells and known to reflect an acute phase of neuronal damage and glial activation, by exponentially increasing their expressions in the brain and bloodstream. However, because the vast majority of these serological factors, with NF-L as an exception (16, 17), have short plasma half-lives (<6 h) due to protease degradation and filtrations by kidney and liver (7), there are significant limitations in their prognostic value and longitudinal utility after concussions.

To overcome these limitations, cellular vesicles such as exosomes have emerged as a novel means of detecting a wide array of diseases in oncology, cardiovascular, and neuroscience communities, given that exosomes are continuously detectable from cells undergoing activation, injury, inflammation, or infection (18–20). In the present study, we captured a double-positive signal containing CD81^+^, exclusively expressed on the surface of extracellular vesicles such as exosomes (21, 22), and cell-specific antigens for neuron, astrocyte, oligodendrocyte, and microglia, respectively. We first examined the test–retest reliability of the panel of serological signals containing both CD81^+^ and cellular factors from neurons, astrocytes, oligodendrocytes, and microglia in healthy individuals at three time points in 2 weeks. Second, changes in the biomarker profile between pre- and post-season were evaluated in professional ice hockey players, aiming to assess neural burden from a single hockey season. Finally, the biomarker patterns from the two players with concussions who showed highly different recovery time courses and profiles were reported.

## Materials and Methods

### Experimental Design and Participants

For the reliability study, we collected blood samples once a week for three times from six healthy adults (four females and two males; age: 31.5 ± 15.1 years) who have no history of brain injury or neurological defects. For the concussion study, we recruited eight professional ice hockey players (eight males; age: 26.63 ± 1.6 years; body mass index: 25.97 ± 1.65 k/m^2^) and a pair of plasma samples from pre-season baseline and post-season follow-up of the 2015–2016 ice hockey season was obtained. During the course of the season, two concussion cases were identified: subject 3 (concussion case #1) with a complete symptom resolution by 7 days after concussion, while subject 6 (concussion case #2) persisted his concussion symptoms until 36 days after concussion (2). Plasma samples along with clinical data were collected until full return-to-play with no symptoms. For the concussion case #1, data points were at 3, 9, and 17 days post-concussion as well as post-season time point corresponding to 130 days post-concussion. For the concussion case #2, data points were at 1, 9, 16, 22, 29, 36, 43, and 50 days post-concussion as well as post-season time point corresponding to 57 days post-concussion. The University of Louisiana Lafayette Institutional Review Board approved the study, and the participants gave written informed consent.

### Clinical Assessments

Near point of convergence (NPC) was assessed based on our established protocol (23, 24). Briefly, the accommodative ruler (Gulden Ophthalmics, Elkins Park, PA, USA) rested on the player’s upper lip, and an accommodative target (reduced-size Snellen chart) was moved down the length of the ruler toward the eyes at a rate of approximately 1–2 cm/s. NPC was taken when the tester observed eye misalignment or when participants verbally signaled experiencing diplopia. On verbal signal, the tester stopped moving the target and recorded the distance between the participant and object. Test was repeated twice, and the average value was used for analysis.

Balance error scoring system (BESS) is a valid series of three balance tasks (25). These tasks were performed first on a firm surface and standardized close-cell foam mat (Airex AG, Switzerland). The three-balance tasks included 2 ft together stance, single-leg non-dominant side stance, and tandem stance with the dominant foot forward. The subjects performed these six tasks with their eyes closed over a single 20 s trial. A single tester counted errors during the trial using established criteria (25). Upon completion of the six tasks, a floor and mat score was calculated for each player. Both NPC and BESS data were obtained by one trained individual for all participants, including all time points from the concussed players.

### Blood Sampling of Healthy Participants and Ice Hockey Players

For both the reliability and concussion studies, 10 mL of venous blood was drawn into vacutainer sterile tubes containing 0.5 mL saline with EDTA and centrifuged for 15 min at 2,500 × *g* and 4°C. Plasmas were stored in 0.5 mL aliquots at −80°C.

### ELISA Detection and Quantification of Serological Factors

Monoclonal antibodies were biotinylated by mixing with EZ-Link Sulfo-NHS-LC-Biotin (Thermo Fisher Scientific) at room temperature for 30 min, followed by the purification of antibodies by spin columns, according to the instruction manual of the product. We selected the following plain monoclonal antibodies against neuron-specific proteins [synaptosome-associated protein 25 (SNAP25), NF-L, tau, and SYP], astrocyte-specific proteins (EAAT1 and GFAP), oligodendrocyte-specific proteins (OMG MBP), microglia–macrophage-specific protein (CD11b), and cytokines [interleukin 8 (IL8) and tumor necrosis factor alpha (TNFα)] (Santa Cruz Biotechnology, Dallas, TX, USA), as well as exosome-enriched marker CD81 (LSBio, Seattle, WA, USA).

The principle of our sandwich ELISA is to capture neuron-, astrocyte-, oligodendrocyte-, and microglia/macrophage-derived materials directly from plasma on the ELISA wells, where anti-SNAP25, anti-EAAT1, anti-OMG, and anti-CD11b antibodies (Santa Cruz Biotechnology, Dallas, TX, USA) are immobilized, respectively. Plasma samples were further reacted with biotinylated antibody against CD81 (LSBio). Our targets were double-positive signals, [CD81]_SNAP25+_ for neuronal origin, [CD81]_EAAT1+_ for astrocyte origin, [CD81]_OMG+_ for oligodendrocyte origin, and [CD81]_CD11b+_ for microglia origin. Separately, SNAP25 plates were reacted with biotinylated antibodies against various neuronal proteins (NF-L, tau) as well as cytokines (TNFα and IL8), and we named them [NF-L]_SNAP25+_, [Tau]_SNAP25+_, [TNFα]_SNAP25+_, and [IL8]_SNAP25+_, respectively. Similarly, EAAT1 plates and OMG plates were reacted with biotinylated antibodies against astrocyte-specific protein GFAP and oligodendrocyte-specific protein MBP, respectively, and the results are named as [GFAP]_EAAT1+_ and [MBP]_OMG+_. Anti-CD11b antibody is expressed in microglia as well as peripheral blood macrophages. Yet, double-positive signals of both CD11b and neuron-specific protein, SYP, suggest microglial phagocytosis of damaged neurons, named as [SYP]_CD11b+_. These markers were normalized by [CD81]_SNAP25+_ for neuronal double-positive markers (i.e., [NF-L]_SNAP25+_), [CD81]_EAAT1+_ for astrocyte ([GFAP]_EAAT1+_), [CD81]_OMG+_ for oligodendrocyte([MBP]_OMG+_), or [CD81]_CD11b+_ for microglia ([SYP]_CD11b+_) markers. For instance, dividing [NF-L]_SNAP25+_ by neuron-related exosomes [CD81]_SNAP25+_ yields the values of NF-L per neuron-related exosome. In other words, non-normalized values ([NF-L]_SNAP25+_) are the amount in plasma, and normalized values ([NF-L]_SNAP25+_/[CD81]_SNAP25+_) are the amount per neuron-related exosome.

Various antibodies were suspended in 1X coating buffer and applied to white eight-well strips in a final volume of 50 µL. After 1 h of incubation at room temperature, each well was washed once with 1X wash buffer, then 75 µL of blocker casein solution supplemented with 0.5% BSA (Equitech) was added into each well for blocking. After another 1 h of incubation at room temperature, each well was washed twice with 1X wash buffer, then 40 µL of plasma samples was added into each well. Plasma was diluted in PBS without any detergent and incubated in a refrigerator overnight. Next day, each well was washed twice with 1X wash buffer, then 40 µL of biotinylated antibody solution supplemented with 4 µg/mL mouse IgG and 1% BSA (Thermo Fisher Scientific) in 0.1% tween 20-PBS was added, and incubation was continued at room temperature for 1 h. After washing, 40 µL of SA-HRP supplemented with 5% blocker casein and 0.25% BSA (Equitech) in 0.1% tween 20-PBS was added, and incubation was continued at room temperature for the final 30 min. After washing, 50 µL of SuperSignal (Thermo Fisher Scientific) was added, and chemiluminescent signals [relative light units (RLU)] were determined in a luminometer (ANSH Labs, Webster, TX, USA).

To quantify the signals, we assigned 100 U/mL to a positive control sample, a dilution study was conducted in each ELISA to obtain RLU in each dilution. Then, using four-parameter logistic analysis, the RLU of each sample was converted to units per millilitre.

### Protein Biomarker Assay

Plasma samples from two concussion cases were analyzed for protein biomarkers including tau, NF-L, GFAP (Millipore, Billerica, MA, USA), TNFα, and IL8 (Quanterix, Lexington, MA, USA) using commercially available ELISA kits. Fluorescent signals measured by a microplate reader (BioTek EL800, Winooski, VT, USA) were converted into ng/mL as per standard curve concentrations. The experimenter performing the assay was blinded from subject information.

### Statistical Analyses

A series of Shapiro–Wilk tests revealed that a data set was not well-modeled by a normal distribution. Therefore, Friedman tests were conducted to examine a stability of biomarker expressions, followed by an individual intraclass correlation coefficient (ICC) to assess test–retest reliabilities across three time points (26). Using a two-way mixed-effects analysis of variance model, we estimated the ICC for baseline (day 0) to week 1 (day 7) and for week 1 (day 7) to week 2 (day 14). ICC is conceptually positive between 0 (not reliable at all) and 1 (perfectly consistent between repeated measurements), but its estimation can be negative in a few cases. We put the negative ICC values to be zeros as commonly done by various studies (27, 28). Based on the value of ICC, reliability is often categorized as poor (ICC = 0–0.2), fair (0.2–0.4), moderate (0.4–0.6), substantially good (0.6–0.8), and excellent (>0.8) (29). Next, Wilcoxon signed-rank tests were used to evaluate the changes in biomarker levels between pre-season baseline and post-season follow-up in professional ice hockey players. All data analyses were conducted using SPSS (version 25.0; SPSS Inc., Chicago, IL, USA), and statistical significance was set at *a* ≤ 0.05.

## Results

### Standard Curves

ELISA readings (RLU) of each biomarker level were successfully converted to units per millilitre by using standard curves yielded from the preliminary dilution study, as illustrated in Figure S1 in Supplementary Material.

### Fluctuation of Plasma Levels of Biomarker Expressions

Levels of all biomarkers were widely spread among six healthy subjects over 2–3 logs. After normalization by [CD81]_SNAP25+_ for neuronal-, [CD81]_EAAT1+_ for astrocyte-, [CD81]_OMG+_ for oligodendrocyte-, or [CD81]_CD11b+_ for microglia-related markers, such variations reduced to 1–2 logs (Figures S2 and S3 in Supplementary Material). Friedman tests showed no statistically significant difference between each time point. The test–retest reliability across three time points showed an excellent agreement for virtually all markers (Table 1), indicating a minimum within-subject fluctuation over time.

**Table 1:** Stability of biomarker levels across three time points.

Markers	χ*^2^*	*P*	Baseline to day 7		Day 7 to day 14	
			ICC (95% CI)	*P*	ICC (95% CI)	*P*
[CD81]SNAP25^+^	4.000	0.135	0.902 (0.389, 0.986)	0.013	0.883 (0.328, 0.983)	0.014
[NF-L]_SNAP25+_	1.249	0.328	0.989 (0.908, 0.998)	<0.001	0.980 (0.879, 0.997)	<0.001
[Tau]_SNAP25+_	0.783	0.676	0.992 (0.949, 0.999)	<0.001	0.971 (0.810, 0.996)	0.001
[TNFα]_SNAP25+_	0.333	0.846	0.998 (0.985, 1.000)	<0.001	0.996 (0.974, 0.999)	<0.001
[IL8]_SNAP25+_	0.333	0.846	0.995 (0.969, 0.999)	<0.001	0.985 (0.903, 0.998)	<0.001
[NF-L]_SNAP25+_/[CD81]_SNAP25+_	3.037	0.094	0.974 (0.556, 0.997)	<0.001	0.948 (0.462, 0.993)	0.001
[Tau]_SNAP25+_/[CD81]_SNAP25+_	5.333	0.069	0.832 (0, 0.977)	0.048	0.729 (0, 0.960)	0.024
[TNFα]_SNAP25+_/[CD81]_SNAP25+_	4.333	0.115	0.829 (0, 0.976)	0.015	0.729 (0, 0.959)	0.050
[IL8]_SNAP25+_/[CD81]_SNAP25+_	4.000	0.135	0.947 (0.685, 0.992)	0.003	0.944 (0.425, 0.993)	0.001
[CD81]_EAAT1+_	0.333	0.846	0.759 (0, 0.965)	0.071	0.784 (0, 0.968)	0.043
[GFAP]_EAAT+_	2.333	0.311	0.923 (0.545, 0.989)	0.006	0.893 (0.367, 0.985)	0.010
[GFAP]_EAAT1+_/[CD81]_EAAT1+_	0.333	0.846	0.897 (330, 0.985)	0.015	0.969 (0.776, 0.996)	0.001
[CD81]OMG^+^	1.708	0.230	0.900 (0.378, 0.986)	0.013	0.845 (0.132, 0.977)	0.023
[MBP]_OMG+_	4.000	0.135	0.971 (0.825, 0.996)	0.001	0.988 (0.931, 0.998)	<0.001
[MBP]_OMG+_/[CD81]_OMG+_	5.695	0.095	0.713 (0, 0.957)	0.080	0.817 (0, 0.974)	0.014
[SYP]_CD11b+_/[CD81]_CD11b+_	0.333	0.846	0.915 (0.481, 0.988)	0.009	0.922 (0.528, 0.989)	0.005

*χ^2^ values and corresponding P values were obtained from Friedman tests. Intraclass correlation coefficient was conducted to examine test–retest reliabilities of each biomarker*.

*CI, confidence interval; SNAP25, synaptosome-associated protein 25, NF-L, neurofilament light polypeptide; TNFα, tumor necrosis factor-alpha; IL8, interleukin-8; EAAT1, excitatory amino acid transporter 1; GFAP, glial fibrillary acidic protein; OMG, oligodendrocyte myelin glycoprotein; MBP, myelin basic protein; SYP, synaptophysin*.

### Changes of Biomarkers Before and After Ice Hockey Season

Wilcoxon signed-rank test indicated that there was a significant increase in [NF-L]_SNAP25+_/[CD81]_SNAP25+_ at post-season compared to pre-season baseline (*Z* = −2.100, *P* = 0.036); however, the statistical significance did not withstand Bonferroni correction for multiple comparisons. All other markers also failed to reach statistical significance (Table 2). Individual expression levels are listed in Table 3. It is worth noting that subject 1, who had four previous concussions, showed greater than a threefold increase in neuronal markers ([NF-L]_SNAP25+_/[CD81]_SNAP25+_, [Tau]_SNAP25+_/[CD81]_SNAP25+_, [TNFα]_SNAP25+_/[CD81]_SNAP25+_, and [IL8]_SNAP25+_/[CD81]_SNAP25+_), as well as oligodendrocyte markers ([MBP]_OMG+_/[CD81]_OMG+_). Subject 4, who had one previous concussion, also showed more than a 2.5-fold increase in neuronal markers (Table 3).

**Table 2:** Changes in exosome markers between pre- and post-season.

Exosome markers	Pre-season (*n* = 8)	Post-season (*n* = 8)
[CD81]SNAP25^+^	4.39 (7.72)	3.22 (4.54)
[NF-L]_SNAP25+_/[CD81]_SNAP25+_	12.58 (11.89)	21.65 (15.90)
[Tau]_SNAP25+_/[CD81]_SNAP25+_	3.25 (2.07)	4.60 (2.91)
[TNFα]_SNAP25+_/[CD81]_SNAP25+_	10.75 (10.78)	16.30 (12.04)
[IL8]_CD81+SNAP25+_/[CD81]_SNAP25+_	13.68 (13.34)	23.64 (19.68)
[CD81]_EAAT1+_	6.56 (10.04)	7.12 (12.32)
[GFAP]_EAAT1+_/[CD81]_EAAT1+_	5.89 (9.43)	4.59 (4.34)
[CD81]_OMG+_	8.36 (11.56)	8.42 (13.27)
[MBP]_OMG+_/[CD81]_OMG+_	15.61 (20.74)	25.49 (28.18)
[SYP]_CD11b+_/[CD81]_CD11b+_	40.40 (31.85)	45.66 (46.70)

*Mean units per millilitre (SD)*.

*Results showed no significant difference in all markers between pre- and post-season*.

*SNAP25, synaptosome-associated protein 25, NF-L, neurofilament light polypeptide; TNFα, tumor necrosis factor-alpha; IL8, interleukin-8; EAAT1, excitatory amino acid transporter 1; GFAP, glial fibrillary acidic protein; OMG, oligodendrocyte myelin glycoprotein; MBP, myelin basic protein; SYP, synaptophysin*.

**Table 3:** Individual profiles of biomarker expressions between pre- and post-season (dark shade, drastic increase possibly due to brain trauma; gray shade, increase not related to brain trauma).

	Concussion		[CD81]_SNAP25+_			[NF-L]_SNAP25+_/[CD81]_SNAP25+_			[Tau]_SNAP25+_/[CD81]_SNAP25+_			[TNFα]_SNAP25+_/[CD81]_SNAP25+_			[IL8]_SNAP25+_/[CD81]_SNAP25+_		
						
n	Previous	This season	Pre	Post	Fold	Pre	Post	Fold	Pre	Post	Fold	Pre	Post	Fold	Pre	Post	Fold
1	4	0	2.38	1.75	0.73	6.85	45.4	**6.63**	1.77	6.01	**3.39**	4.89	33.1	**6.76**	6.10	58.2	**9.54**
2	0	0	0.24	0.61	**2.51**	6.21	15.5	2.49	2.36	3.63	1.54	9.66	12.8	1.33	10.7	17.1	1.60
3	2	1	23.2	13.4	0.58	2.86	6.40	2.24	2.15	2.43	1.13	2.30	3.92	1.71	2.42	4.76	1.97
4	1	0	0.54	0.62	1.16	10.8	27.6	**2.56**	2.49	7.60	**3.05**	9.30	22.2	2.38	12.0	31.3	**2.61**
5	0	0	0.06	0.15	**2.74**	39.3	43.8	1.12	7.90	9.88	1.25	36.0	33.4	0.93	43.9	45.4	1.03
6	1	1	2.31	1.05	0.46	14.6	14.1	0.96	4.50	2.21	0.49	9.85	10.0	1.02	14.6	13.9	0.95
7	2	0	4.28	6.29	1.47	3.38	3.48	1.03	1.74	1.99	1.14	2.58	2.68	1.04	2.52	3.04	1.21
8	0	0	2.16	1.86	0.86	16.7	16.9	1.01	3.08	3.09	1.00	11.5	12.3	1.07	17.2	15.5	0.90

	**Concussion**		**[CD81]_EAAT1+_**			**[GFAP]_EAAT1+_/[CD81]_EAAT1+_**			**[CD81]OMG^+^**			**[MBP]_OMG+_/[CD81]_OMG+_**			**[SYP]_CD11b+_/[CD81]_CD11b+_**		
						
**n**	**Previous**	**This season**	**Pre**	**Post**	**Fold**	**Pre**	**Post**	**Fold**	**Pre**	**Post**	**Fold**	**Pre**	**Post**	**Fold**	**Pre**	**Post**	**Fold**

1	4	0	1.83	1.16	0.63	2.42	4.79	1.98	2.78	1.81	0.65	8.29	88.9	**10.73**	36.3	48.5	1.34
2	0	0	0.21	0.68	**3.28**	4.34	3.20	0.74	0.46	1.42	**3.06**	14.0	10.5	0.75	8.48	18.5	2.19
3	2	1	27.2	16.2	0.60	0.85	1.26	1.49	32.3	19.5	0.60	5.08	10.3	2.02	77.7	156	2.01
4	1	0	0.81	1.02	1.25	2.64	4.88	1.85	1.42	1.73	1.22	8.76	16.0	1.82	16.6	30.0	1.80
5	0	0	0.03	0.14	**4.11**	29.0	14.5	0.50	0.07	0.27	**4.16**	66.0	34.9	0.53	6.20	10.3	1.66
6	1	1	2.72	0.60	0.22	3.90	4.67	1.20	4.83	0.69	0.14	13.2	32.9	2.50	89.5	31.6	0.35
7	2	0	17.1	34.6	2.02	0.41	0.26	0.64	19.8	37.3	1.88	1.67	1.33	0.80	28.4	20.4	0.72
8	0	0	2.61	2.53	0.97	3.64	3.11	0.86	5.20	4.67	0.90	7.88	9.16	1.16	60.0	50.2	0.84

*Unit in units per millilitre for [CD81]_SNAP25+_, [CD81]_EAAT1+_, and [CD81]_OMG+_. Unit for rest of the biomarkers are in ratio. Unit in ratio*.

*SNAP25, synaptosome-associated protein 25, NF-L, neurofilament light polypeptide; TNFα, tumor necrosis factor-alpha; IL8, interleukin-8; EAAT1, excitatory amino acid transporter 1; GFAP, glial fibrillary acidic protein; OMG, oligodendrocyte myelin glycoprotein; MBP, myelin basic protein; SYP, synaptophysin*.

### Reports of two Concussion Cases

#### Case 1: Transient Defects

The concussion case 1 (subject 3) sustained a concussion in-game from a hockey puck traveling at a high rate of speed that struck him near the chin on his goalie mask. This initial blow caused him to fall backwards toward the goal and hit the back of his head on the crossbar. He lost his consciousness for 15–20 s. The patient regained consciousness shortly after the athletic trainer arrived. During the transport on a spine board, he again lost consciousness for 15–20 s. Diagnostics, including MRI and CT imaging, were performed with no substantial findings, and he was released later that evening.

**Figure 1:**
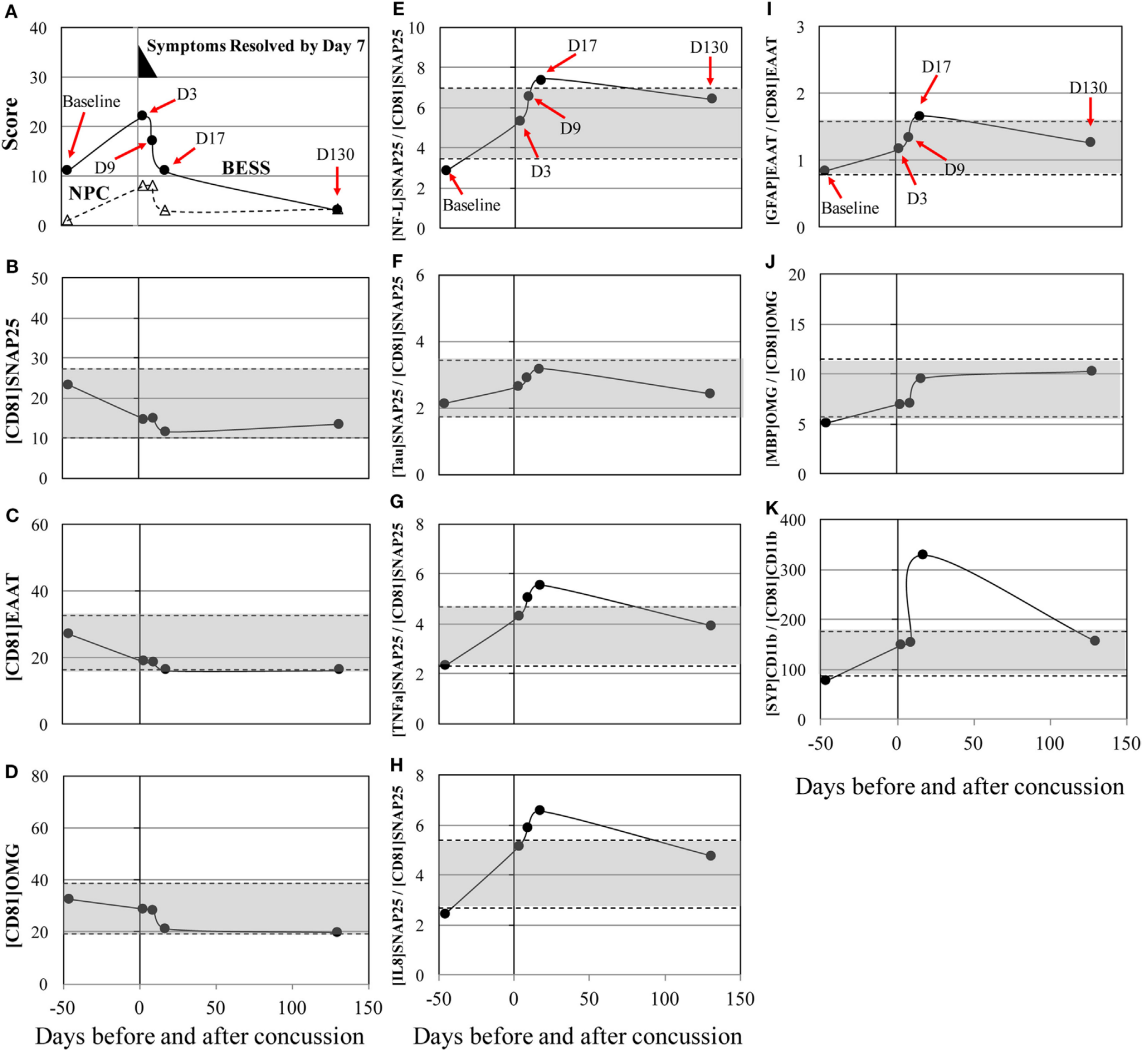
Concussion case 1: transient symptoms. *X* axis is days before and after concussion, with day 0 being the day of concussion. **(A)**: clinical assessment of balance error scoring system (BESS) (●) and near point of convergence (NPC) (Δ). **(B)**: [CD81]_SNAP25+_ (U/mL), **(C)**: [CD81]_EAAT1+_ (U/mL), **(D)**: [CD81]_OMG+_ (U/mL), **(E)**: [NF-L]_SNAP25+_/[CD81]_SNAP25+_ ratio, **(F)**: [Tau]_SNAP25+_/[CD81]_SNAP25+_ ratio, **(G)**: [TNFα]_SNAP25+_/[CD81]_SNAP25+_ ratio, **(H)**: [IL8]_SNAP25+_/[CD81]_SNAP25+_ ratio, **(I)**: [GFAP]_EAAT1+_/[CD81]_EAAT1+_ ratio, **(J)**: [MBP]_OMG+_/[CD81]_OMG+_ ratio, and **(K)**: [SYP]_CD11b+_/[CD81]_CD11b+_.±50% from pre- and post-season average values was shown in shaded blanket as referential values. SNAP25, synaptosome-associated protein 25, NF-L, neurofilament light polypeptide; TNFα, tumor necrosis factor-alpha; IL8, interleukin-8; EAAT1, excitatory amino acid transporter 1; GFAP, glial fibrillary acidic protein; OMG, oligodendrocyte myelin glycoprotein; MBP, myelin basic protein; SYP, synaptophysin.

Post-concussion patterns observed in subject 3 was consistent with current consensus, where 85–90% of concussion patients resolve symptoms and normalize clinical tests to their baseline levels within 1–2 weeks (2, 30, 31). His symptoms dissipated by day 7, but increased errors in BESS and worsening in NPC were observed through days 3–9 after concussion and normalized to the baseline level by day 17 (Figure 1A). Circulating biomarkers, on the other hand, have shown a different pattern from the clinical measures. The levels of [NF-L]_SNAP25+_/[CD81]_SNAP25+_, [TNFα]_SNAP25+_/[CD81]_SNAP25+_, and [IL8]_SNAP25+_/[CD81]_SNAP25+_ (Figures 1E,G,H) slowly increased up to day 17 after concussion. The level of [SYP]_CD11b+_/[CD81]_CD11b+_ (Figure 1K) showed a 422% increase at day 17 after concussion compared to the baseline. All the other markers showed an unremarkable pattern (Figures 1B–D, F, I, J).

#### Case 2: Persisted Symptoms

**Figure 2:**
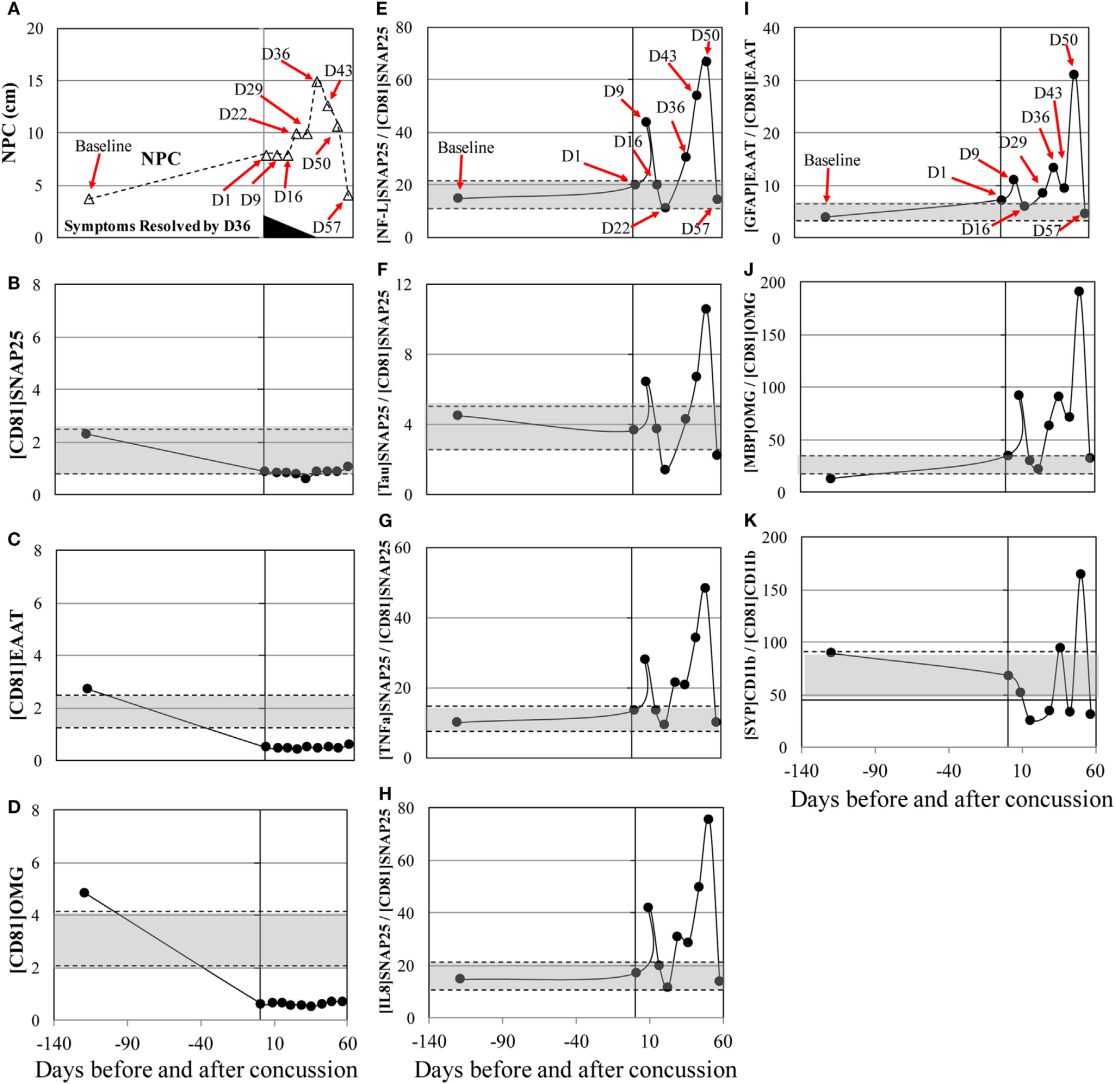
Concussion case 2: persisted symptoms. *X* axis is days before and after concussion, with day 0 being the day of concussion. **(A)**: clinical assessment of near point of convergence (NPC) (Δ).**(B)**: [CD81]_SNAP25+_ (U/mL), **(C)**: [CD81]_EAAT1+_ (U/mL), **(D)**: [CD81]_OMG+_ (U/mL), **(E)**: [NF-L]_SNAP25+_/[CD81]_SNAP25+_ ratio, **(F)**: [Tau]_SNAP25+_/[CD81]_SNAP25+_ ratio, **(G)**: [TNFα]_SNAP25+_/[CD81]_SNAP25+_ ratio, **(H)**: [IL8]_SNAP25+_/[CD81]_SNAP25+_ ratio, **(I)**: [GFAP]_EAAT1+_/[CD81]_EAAT1+_ ratio, **(J)**: [MBP]_OMG+_/[CD81]_OMG+_ ratio, and **(K)**: [SYP]_CD11b+_/[CD81]_CD11b+_. +50% from pre- and post-season average values was shown in shaded blanket as referential values. SNAP25, synaptosome-associated protein 25, NF-L, neurofilament light polypeptide; TNFα, tumor necrosis factor-alpha; IL8, interleukin-8; EAAT1, excitatory amino acid transporter 1; GFAP, glial fibrillary acidic protein; OMG, oligodendrocyte myelin glycoprotein; MBP, myelin basic protein; SYP, synaptophysin.

The concussion case 2 (subject 6) sustained a mild, glancing blow to the right temporal area of the head during a slow pace practice drill. The patient had no loss of consciousness and mild symptoms initially. Over the next few days, the patient began to develop a significant headache, balance issues during activities of daily living, and an aversion to loud, sudden noises. His concussion symptoms—particularly, headache and sensitivity to noise—persisted for more than a month and resolved at day 36 after concussion. NPC slowly increased and peaked at day 36 after concussion (Figure 2A) and returned to the baseline levels by day 57. MRI at day 36 showed no abnormality. Substantial biphasic increases, peaked at day 50, were observed in [NF-L]_SNAP25+_/[CD81]_SNAP25+_, [Tau]_SNAP25+_/[CD81]_SNAP25+_, [TNFα]_SNAP25+_/[CD81]_SNAP25+_, and [IL8]_SNAP25+_/[CD81]_SNAP25+_ (Figures 2E–K). Such drastic changes in the neuronal markers reflected throughout post-concussion recovery period, while imaging analysis (MRI) failed to detect any changes.

### Analysis of Protein-Based Biomarkers in two Concussion Cases

**Figure 3:**
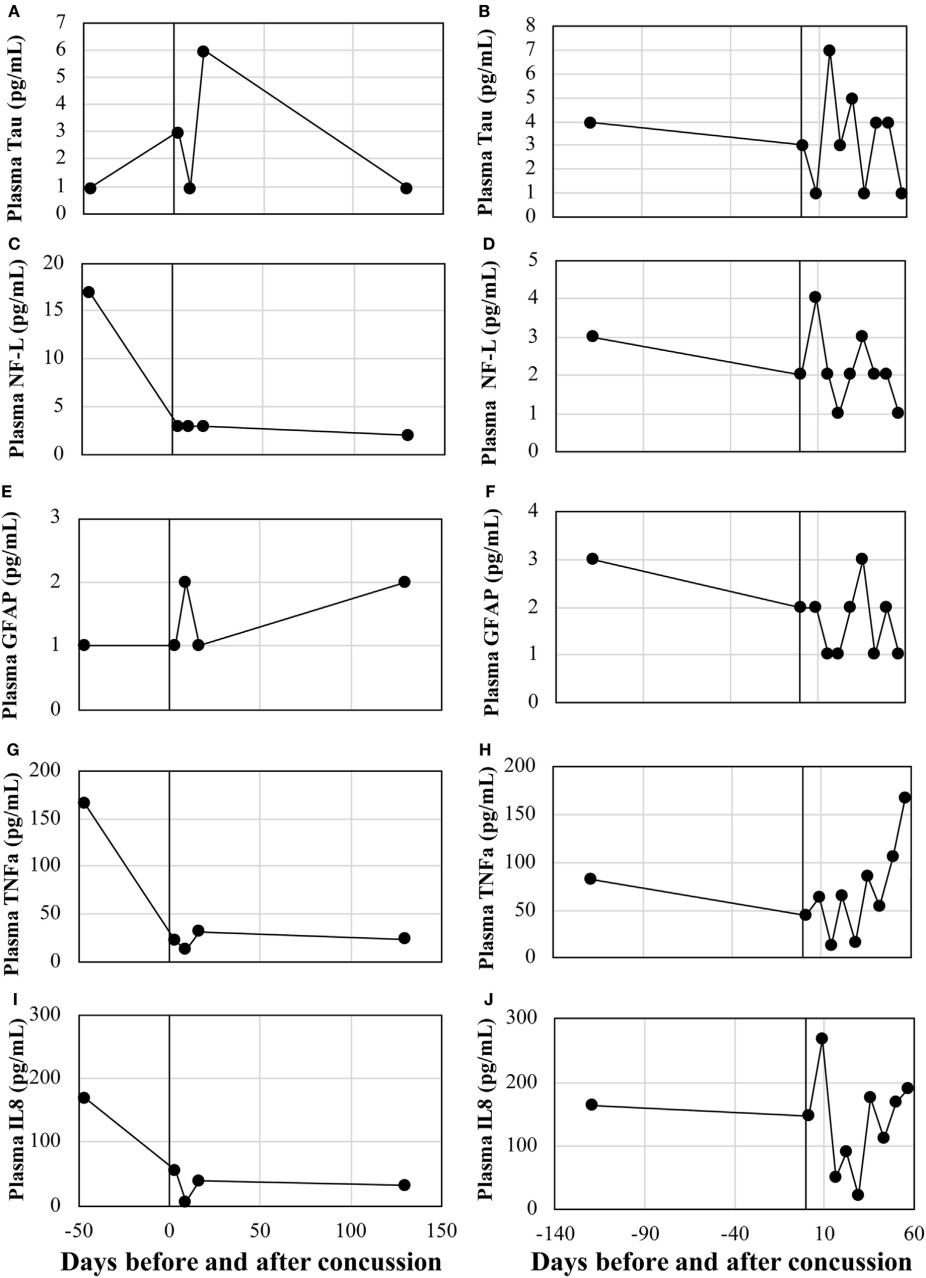
Serum levels of conventional protein biomarkers. Case 1: **(A)** Tau, **(B)** NF-L, **(C)** GFAP, **(D)** TNFα, and **(E)** IL8. Case 2: **(F)** Tau, **(G)** NF-L, **(H)** GFAP, **(I)** TNFα, and **(J)** IL8. NF-L, neurofilament light polypeptide; TNFα, tumor necrosis factor-alpha; IL8, interleukin-8; GFAP, glial fibrillary acidic protein.

Circulating protein expressions of tau, NF-L, GFAP, TNFα, and IL8 were assessed in the two concussion cases. There was no remarkable trend for all biomarkers, with the expression levels being widely fluctuated across recovery time points (Figure 3).

## Discussion

We reported unique profiles of blood biomarkers for two cases of concussion as well as pre- and post-season changes in professional ice hockey players. The major findings of the present studies are that an array of biomarkers including neuronal- and microglia origin increased after concussions, with the unique expression patterns being similar to that of concussion recovery process. Excellent test–retest reliabilities of these markers further underpin that the robust increases observed in concussed individuals are potentially due to the damage in neural cell structures and metabolic crisis. To our knowledge, this is the first case report demonstrating an array of blood biomarkers to gradually raise in concert with clinical measures in two of the concussion cases.

To date, there are no biomarkers including NF-L, tau, TNFα, IL8, and SYP that are useful in a longitudinal approach because of their relatively short plasma half-lives. To overcome this major limitation, we targeted serological factors that are reacted positive with CD81 and various neuron-specific and inflammatory antigens. It is highly plausible that the detected signals are circulating exosomes containing neuron- and glia-specific factors on their surface. Exosomes (30–100 nm in diameter) are a mode of intercellular communication that shuttles vesicles containing bioactive molecules (e.g., proteins, mRNA, and microRNA) from one cell to another through interstitial fluid for proximal cells and the blood stream for distant recipients (32). During the genesis of exosomes, portions of the plasma membrane are internalized as endosomes or intraluminal endosomal vesicles within the host cells. Bioactive products are packed inside the vesicles, while numerous cytosolic proteins adhere on the surface of the vesicles. These vesicles become exosomes when they merge with the cell membrane and release into the extracellular space (33).

The increases in NF-L and Tau markers suggest axonal injury, whereas the increases in TNFα and IL8 markers are due to neuroinflammation. The increases in GFAP and MBP markers are indicative of the activation of astrocytes and oligodendrocytes as a part of cellular repairing process, as well as apoptotic cell death in glial cells. The elevation in [SYP]_CD11b+_/[CD81]_CD11b+_ means that microglia phagocytosis damaged neurons, generating microglia-derived exosomes containing neuron-specific marker, synaptophysin. The changes in biomarker levels between pre- and post-season are worthy of note, although all markers did not reach statistical significance due to its small sample size and after Bonferroni correction to account for multiple comparison. One potential reason that there was unremarkable increase in the post-season biomarker data of the concussion subjects (#3 and 6) was because these players sufficiently rested without incurring subconcussive head impacts. Particularly for the case 2, his concussion occurred toward the end of the season. His post-season data point corresponds to the 57-day post-concussion time point. During this recovery time, he did not participate in any activity involving head impacts. Therefore, his post-season biomarker values were normalized to the baseline level after resting for close to 2 months (Figure 2). On the contrary, his teammates (*n* = 7 including concussion case #1) continued to engage in practices and games until the end of season, experiencing head and body impacts. Therefore, albeit statistically non-significant, there was an increase in the absolute value of neuron-related biomarker profile at post-season compared to the pre-season baseline. To examine the effect of subconcussive head impacts, we are currently analyzing the array of our markers in high school football players from 12 different time points in a season, in relation to the frequency and magnitude of head impacts measured by accelerometer-installed mouth guard (23, 34, 35).

There are several key limitations in the study. Our sandwich ELISA assays are not directly measuring exosomes, rather we normalized the double-positive signals specific to brain parenchymal cells by exosome signals. This way, we eliminated a number of steps required to isolate exosomes that are inherent to increased technical errors and cost (36). On the contrary, our sandwich ELISA technique is reliable, time saving, and cost effective, and thus, it is feasible for clinical testing. While results from the aforementioned cases are fascinating, a lack of sample size and data regarding head impact kinematics during the hockey season substantially hinder our interpretation of increased biomarker levels. Moreover, we did not have blood samples from concussion-free control subjects such as teammates, corresponding to the recovery time points of the concussion subjects. Due to the lack of these samples accounting for the effects of sub-clinical head impacts (e.g., subconcussive head impacts), we were unable to make a meaningful interpretation of the biphasic increase observed in the concussion case 2. Thus, it is imperative to validate the current findings in a larger sample size with appropriate control groups in conjunction with identifying sensitivity and specificity of these panels of biomarkers. Additional limitation is that although the within-subject stability of all exosome markers was excellent, there was between-subject disparity, suggesting the limited applicability for cross-sectional studies. On the contrary, the results have a tremendous implication in longitudinal assessments, where changes in biomarker levels from baseline level can be tracked over time to aid physicians to determine return-to-play/work, establish a threshold for cumulative forces to the head, and monitor the progression of neurodegenerative pathology. Finally, it is well-accepted notion that cerebral spinal fluid (CSF) biomarkers are superior in reflecting the severity and prognostication of brain trauma with higher sensitivity and specificity and longer half-life than those of blood-based biomarkers (37). Brain-derived factors not only cross breached blood–brain barrier into the blood stream but also are drained into subarachnoid space *via* the glymphatic system (7). Fraction of CSF markers is further drained into the blood stream through subclavian veins *via* lymphatic ducts, indicating that elevated biomarker levels in the blood are often derivative of CSF biomarkers. However, because of an invasive nature of the CSF marker assessment, an alternative approach including protein-based and exosome blood biomarkers is feasible in concussion cohort.

In summary, the present study suggests that a unique panel of blood biomarkers that may have the potential to surrogate chronic neural damage caused by sport-related concussion. Elevations in the blood biomarkers in this study were in line with the reports from chronic traumatic encephalopathy, Alzheimer’s disease, and dementia patients (38–40), although remotely at this point. The current study serves as an excellent initial step to employ potential exosome biomarkers as a means to objectively monitor concussed patients’ recovery.

## Ethics Statement

This study was carried out in accordance with the recommendations of The University of Louisiana Lafayette, Institutional Review Board committee with written informed consent from all subjects. All subjects gave written informed consent in accordance with the Declaration of Helsinki. The protocol was approved by The University of Louisiana Lafayette Institutional Review Board.

## Author Contributions

KK and RA conceptualized and designed the study and the data collection instruments, collected data, drafted the initial manuscript, and reviewed and revised the manuscript. RA conceptualized and designed the study, collected data, and reviewed and revised the manuscript. MM conducted the experiments, interpreted the analysis, and critically reviewed the manuscript for important intellectual content. All the authors have read and approved the final manuscript as submitted and agree to be accountable for all aspects of the work.

## Conflict of Interest Statement

KK and RA report no competing financial interests. MM is an employee of NanoSomiX, Inc.

## Supplementary Material

Figure S1 The results of dilution study of standard plasma. ELISA readings (RLU) (*Y*-axis) were converted to units per millilitre (*X*-axis) by assigning the standard plasma as 100 U/mL. **(A)** [CD81]_SNAP25+_, **(B)** [CD81]_EAAT1+_, **(C)** [CD81]_OMG+_, **(D)** [SYP]_CD11b+_, **(E)** [Tau]_SNAP25+_, **(F)** [NF-L]_SNAP25+_, **(G)** [TNFα]_SNAP25+_, **(H)** [IL8]_SNAP25+_, **(I)** [GFAP]_EAAT1+_, and **(J)** [MBP]_OMG+_. SNAP25, synaptosome-associated protein 25, NF-L, neurofilament light polypeptide; TNFα, tumor necrosis factor-alpha; IL8, interleukin-8; EAAT1, excitatory amino acid transporter 1; GFAP, glial fibrillary acidic protein; OMG, oligodendrocyte myelin glycoprotein; MBP, myelin basic protein; SYP, synaptophysin; RLU, relative light units.

Figure S2 Fluctuation of plasma levels of NDE and surface biomarkers on neuronal markers. **(A)** [CD81]_SNAP25+_ (U/mL), **(B)** [NF-L]_SNAP25+_ (U/mL), **(C)** [NF-L]_SNAP25+_/[CD81]_SNAP25+_ ratio, **(D)** [Tau]_SNAP25+_ (U/mL), **(E)** [Tau]_SNAP25+_/[CD81]_SNAP25+_ ratio, **(F)** [TNFα]_SNAP25+_ (U/mL), **(G)** [TNFα]_SNAP25+_/[CD81]_SNAP25+_ ratio, **(H)** [IL8]_CD81+SNAP25+_ (U/mL), and **(I)** [IL8]_SNAP25+_/[CD81]_SNAP25+_ ratio. Each symbol represents a single individual. SNAP25, synaptosome-associated protein 25, NF-L, neurofilament light polypeptide; TNFα, tumor necrosis factor-alpha; IL8, interleukin-8; EAAT1, excitatory amino acid transporter 1; GFAP, glial fibrillary acidic protein; OMG, oligodendrocyte myelin glycoprotein; MBP, myelin basic protein; SYP, synaptophysin.

Figure S3 Fluctuation of plasma levels of glial markers. **(A)** [CD81]_EAAT1+_ (U/mL), **(B)** [GFAP]_EAAT1+_ (U/mL), **(C)** [GFAP]_EAAT1+_/[CD81]_EAAT1+_ ratio, **(D)** [CD81]_OMG+_ (U/mL), **(E)** [MBP]_OMG+_, (U/mL), **(F)** [MBP]_OMG+_/[CD81]_OMG+_ ratio, and **(G)** [SYP]_CD11b+_/[CD81]_CD11b+_. Each symbol represents a single individual. NF-L, neurofilament light polypeptide; TNFα, tumor necrosis factor-alpha; IL8, interleukin-8; EAAT1, excitatory amino acid transporter 1; GFAP, glial fibrillary acidic protein; OMG, oligodendrocyte myelin glycoprotein; MBP, myelin basic protein; SYP, synaptophysin.
