# Supplementary figures and images for: A Preliminary Report on Brain-Derived Extracellular Vesicle as Novel Blood Biomarkers for Sport-Related Concussions
Source: Front Neurol. 2018 Apr 12;9:239. doi: 10.3389/fneur.2018.00239 (PMC5906531; doi:10.3389/fneur.2018.00239)

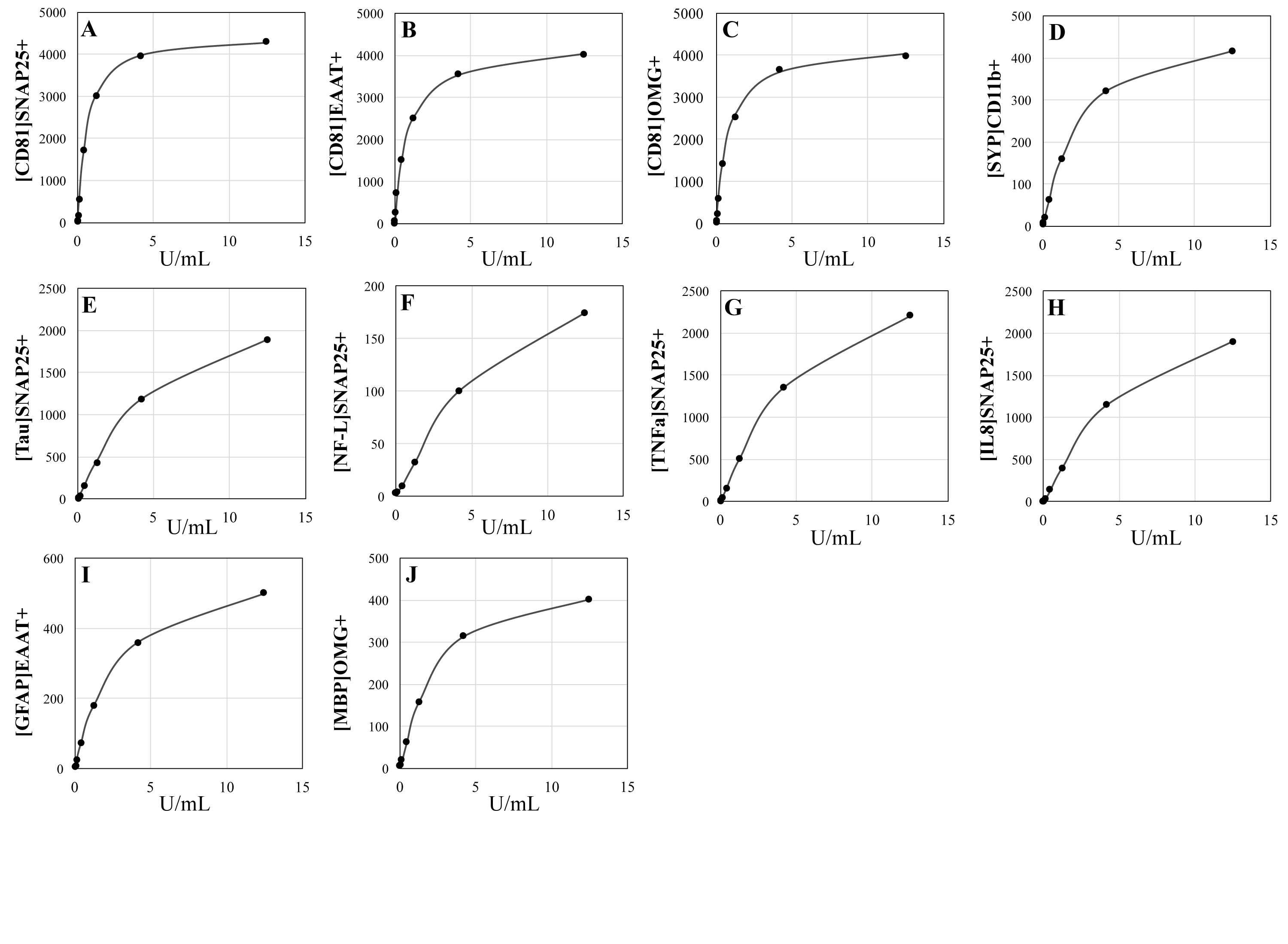

Supplement: Figure S1 — The results of dilution study of standard plasma. ELISA readings (RLU) (Y-axis) were converted to units per millilitre (X-axis) by assigning the standard plasma as 100 U/mL. (A) [CD81]SNAP25+, (B) [CD81]EAAT1+, (C) [CD81]OMG+, (D) [SYP]CD11b+, (E) [Tau]SNAP25+, (F) [NF-L]SNAP25+, (G) [TNFα]SNAP25+, (H) [IL8]SNAP25+, (I) [GFAP]EAAT1+, and (J) [MBP]OMG+. SNAP25, synaptosome-associated protein 25, NF-L, neurofilament light polypeptide; TNFα, tumor necrosis factor-alpha; IL8, interleukin-8; EAAT1, excitatory amino acid transporter 1; GFAP, glial fibrillary acidic protein; OMG, oligodendrocyte myelin glycoprotein; MBP, myelin basic protein; SYP, synaptophysin; RLU, relative light units. [file image_1.tif]

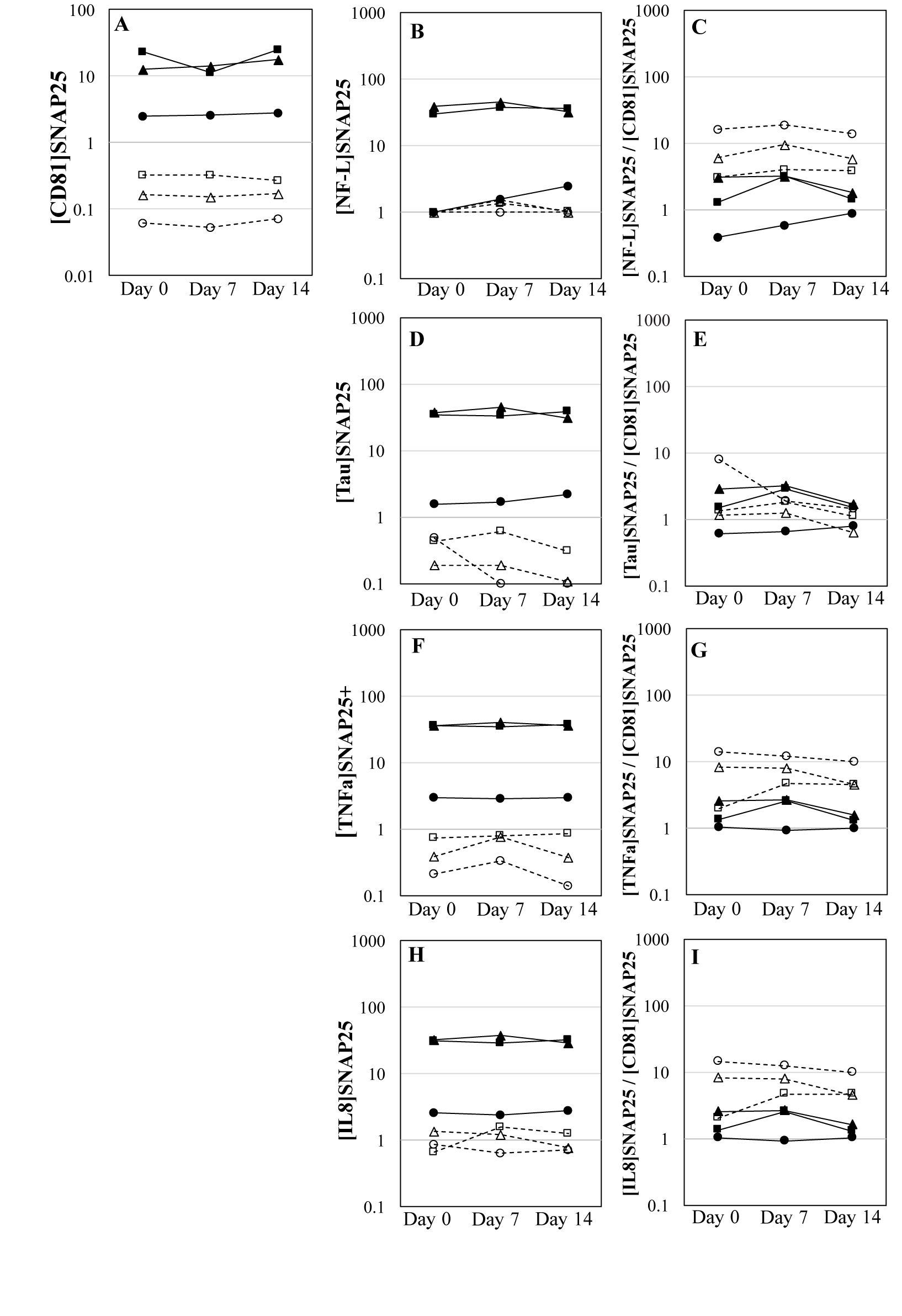

Supplement: Figure S2 — Fluctuation of plasma levels of NDE and surface biomarkers on neuronal markers. (A) [CD81]SNAP25+ (U/mL), (B) [NF-L]SNAP25+ (U/mL), (C) [NF-L]SNAP25+/[CD81]SNAP25+ ratio, (D) [Tau]SNAP25+ (U/mL), (E) [Tau]SNAP25+/[CD81]SNAP25+ ratio, (F) [TNFα]SNAP25+ (U/mL), (G) [TNFα]SNAP25+/[CD81]SNAP25+ ratio, (H) [IL8]CD81+SNAP25+ (U/mL), and (I) [IL8]SNAP25+/[CD81]SNAP25+ ratio. Each symbol represents a single individual. SNAP25, synaptosome-associated protein 25, NF-L, neurofilament light polypeptide; TNFα, tumor necrosis factor-alpha; IL8, interleukin-8; EAAT1, excitatory amino acid transporter 1; GFAP, glial fibrillary acidic protein; OMG, oligodendrocyte myelin glycoprotein; MBP, myelin basic protein; SYP, synaptophysin. [file image_2.tif]

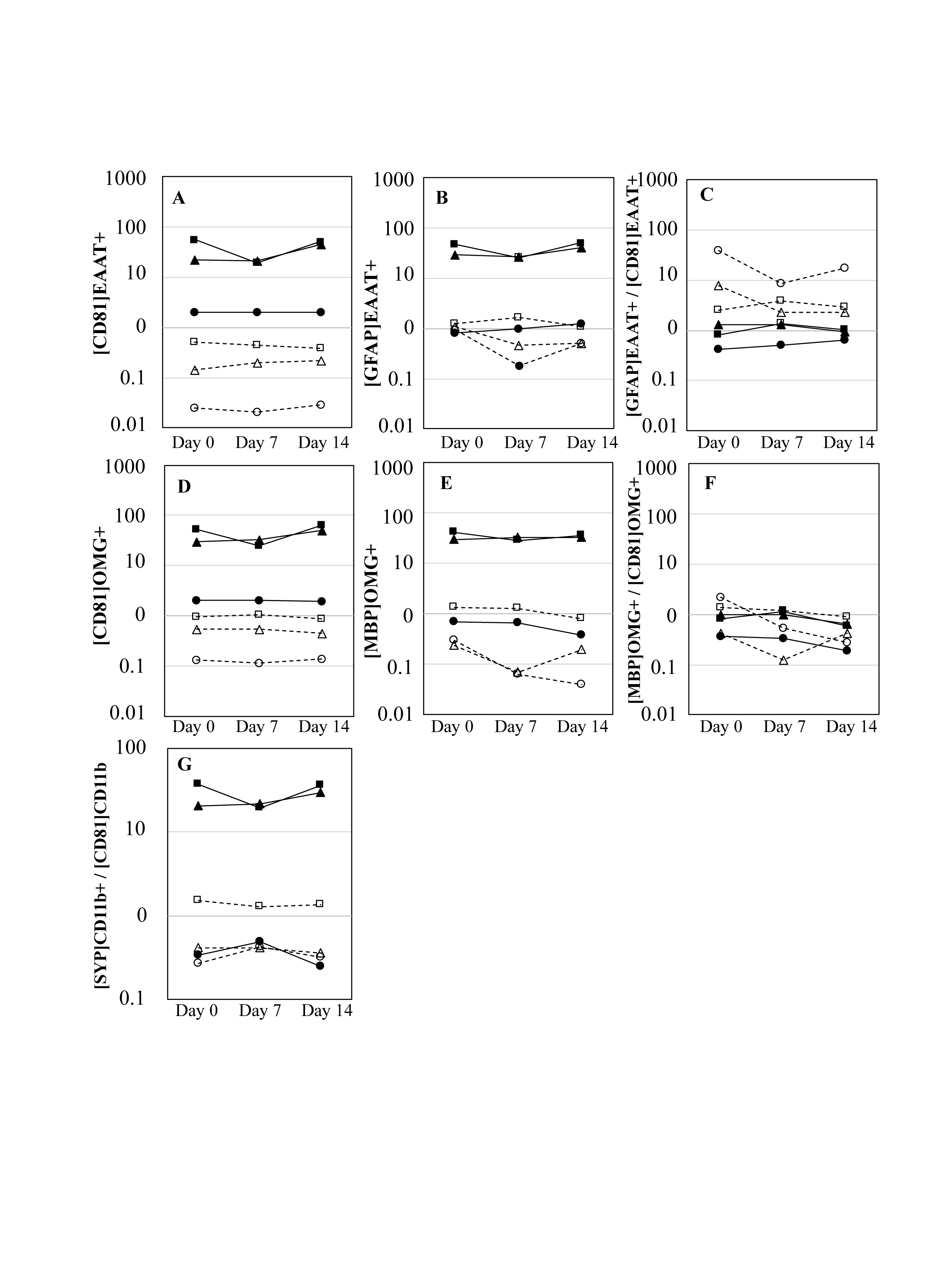

Supplement: Figure S3 — Fluctuation of plasma levels of glial markers. (A) [CD81]EAAT1+ (U/mL), (B) [GFAP]EAAT1+ (U/mL), (C) [GFAP]EAAT1+/[CD81]EAAT1+ ratio, (D) [CD81]OMG+ (U/mL), (E) [MBP]OMG+, (U/mL), (F) [MBP]OMG+/[CD81]OMG+ ratio, and (G) [SYP]CD11b+/[CD81]CD11b+. Each symbol represents a single individual. NF-L, neurofilament light polypeptide; TNFα, tumor necrosis factor-alpha; IL8, interleukin-8; EAAT1, excitatory amino acid transporter 1; GFAP, glial fibrillary acidic protein; OMG, oligodendrocyte myelin glycoprotein; MBP, myelin basic protein; SYP, synaptophysin. [file image_3.tif]
